# Transfer of skills for difficult intubation after videolaryngoscopy training: a randomized simulation study

**DOI:** 10.1186/s13054-020-02982-8

**Published:** 2020-05-25

**Authors:** Adrian Kee, Reyor Ko, Rolando Capistrano, Melvin Dajac, Juvel Taculod, Kay Choong See

**Affiliations:** 1 Division of Respiratory and Critical Care Medicine, National University Hospital, 1E, Kent Ridge Road, NUHS Tower Block, Level 10, Singapore, 119228 Singapore; 2 Department of Medicine, Yong Loo Lin School of Medicine, National University of Singapore, 1E, Kent Ridge Road, NUHS Tower Block, Level 10, Singapore, 119228 Singapore; 3 Ministry of Health Holdings, Singapore, Singapore; 4 Division of Critical Care -- Respiratory Therapy, National University Hospital, Singapore, Singapore

**Keywords:** Videolaryngoscopy, Simulation, Novice, Physician-trainee, Intubation skill

## Research letter

Videolaryngoscopy (VL) is increasingly being used in intensive care units (ICUs) and may increase the chance of first-pass success when intubation is difficult [1–3]. However, VL equipment may not always be available and direct laryngoscopy (DL) would then be required. While previous simulation studies demonstrated comparable retention of skills for DL versus VL in normal manikins [4, 5], it is unknown if VL training among physician trainees would lead to ineffective DL use for difficult intubation scenarios. We therefore aim to show if training using VL would lead to effective transfer of skills for difficult intubation using DL.

Ethical approval was sought from the National Healthcare Group Domain Specific Review Board (DSRB 2015/00937). Internal medicine (IM) residents who had little prior exposure to DL or VL were recruited and randomized into either DL (size 3 Macintosh blade) or VL (C-MAC®) training, using a normal airway manikin (Cormack-Lehane Grade I). For both groups, the intubation method and instructor remained the same. Residents were then assessed by two blinded assessors, using DL on a difficult airway manikin (Cormack-Lehane Grade III). The primary outcome was intubation time during the first attempt (after passing the laryngoscope between the lips), averaged between the two assessors. Intubation time was truncated at 120 s, beyond which the attempt was considered failed. The secondary outcome measures were first pass success rate and rate of complications from ETI (such as teeth damage and endobronchial intubation).

Forty-one residents were randomized (21 DL, 20 VL) (Table 1). The median intubation time taken for the DL and VL groups to intubate were 42.5 s (range 21–120 s) and 41.5 s (range 13–120 s), respectively, *p* = 0.273 (Table 2). Successful intubation on first attempt was recorded in 17 and 18 residents in the DL and VL group, respectively, *p* = 0.542. Between the DL and VL groups, complication rates were not significantly different: teeth damage (5 DL, 4 VL); endobronchial intubation (1 DL, 2 VL). With regards to inter-tester variability, the correlation between 1st and 2nd assessors for participants’ median time for intubation during the first attempt was excellent (Spearman’s rho = 0.992, *p* < 0.001).

**Table 1:** Participant characteristics

Characteristics	All participants (*n* = 41)	DL group (*n* = 21)	VL group (*n* = 20)	*p* value
Age (years)	25.6 ± 2.8	25.0 ± 1.7	26.3 ± 3.6	0.123
Female (%)	19 (46.3)	6 (28.6)	13 (65.0)	0.029
PGY (%)				
1	29 (70.7)	15 (71.4)	14 (70.0)	0.561
2	6 (14.6)	4 (19.1)	2 (10.0)	
> 2	6 (14.6)	2 (9.5)	4 (20.0)	
Medical school (%)				
Local	28 (68.3)	14 (70.0)	14 (66.7)	1.000
Foreign	13 (31.7)	6 (30.0)	7 (33.3)	
Prior ED working experience (%)	4 (9.8)	2 (9.5)	2 (10.0)	1.000
Prior ICU working experience (%)	2 (4.9)	1 (4.8)	1 (5.0)	1.000
Prior number of successful intubations in live patients (%)				
0	23 (56.1)	10 (47.6)	13 (65.0)	0.688
1	9 (22.0)	5 (23.8)	4 (20.0)	
2	1 (2.4)	1 (4.8)	0	
> 2	8 (19.5)	5 (23.8)	3 (15.0)	
Prior intubation training (%)	34 (82.9)	18 (85.7)	16 (80.0)	0.697

*DL* direct laryngoscopy, *ED* emergency department, *ICU* intensive care unit, *PGY* postgraduate year, i.e., number of years after graduation from medical school, *VL* videolaryngoscopy

**Table 2:** Intubation training results and complication rates

Results	All participants (*n* = 41)	DL group (*n* = 21)	VL group (*n* = 20)	*p* value
Timing for 1st attempt (s)^a^				
Median	42	42.5	41.5	0.273
Interquartile range	28–67	38–100	23–59	
Range	13–120	21–120	13–120	
Number of failed attempts (%)				
0	35 (85.4)	17 (81.0)	18 (90.0)	0.542
1	4 (9.8)	2 (9.5)	2 (10.0)	
2	2 (4.9)	2 (9.5)	0	
Other complications (%)				
None	29 (70.7)	15 (71.4)	14 (70.0)	1.000
Teeth damage	9 (22.0)	5 (23.8)	4 (20.0)	
Endobronchial intubation	3 (7.3)	1 (4.8)	2 (10.0)	

^a^Average timing recorded by 1st and 2nd assessors

DL, direct laryngoscopy, *ED* emergency department, *ICU* intensive care unit, *PGY* postgraduate year, i.e., number of years after graduation from medical school, *VL* videolaryngoscopy

In conclusion, training with VL, compared to DL, had similar transfer of skills for difficult intubation using DL. As our randomized trial was done under simulation conditions, further study within an authentic clinical environment would be needed to confirm our preliminary results.

## Data Availability

The datasets generated and/or analyzed during the current study are not publicly available due as the local approval authority does not permit data sets to be placed publicly but are available from the corresponding author on reasonable request.

## Abbreviations

DL: Direct laryngoscopy
VL: Videolaryngoscopy
